# Is There Association between Risky Sexual Behaviors and Depression Symptoms among Youth? A Case of Jimma University Students, Ethiopia

**DOI:** 10.1155/2019/3757656

**Published:** 2019-07-01

**Authors:** Yonas Tesfaye, Alemayehu Negash, Tsegaye Tewelde Gebrehiwot, Worknesh Tessema, Susan Anand, Gutema Ahmed, Daniel Alemu

**Affiliations:** ^1^School of Nursing and Midwifery, Jimma University, Jimma, 378, Ethiopia; ^2^Department of Psychiatry, Jimma University, Jimma, Ethiopia; ^3^Department of Epidemiology and Biostatistics, Jimma University, Jimma, Ethiopia; ^4^Department of Psychiatry, Haramaya University, Harar, Ethiopia

## Abstract

**Background:**

Risky Sexual Behaviors (RSB) and Depression symptoms expose young people to various reproductive health problems including sexually transmitted infections and HIV/AIDS. To date the link between these two major public health problems lacks empirical evidence in the context of higher education institutions in Ethiopia.

**Objective:**

The aim of this study was to assess association between risky sexual behavior and depression symptoms among Jimma University main campus students, Jimma, Ethiopia, 2016.

**Methods:**

An institution based quantitative cross sectional study was conducted. A pre-tested questionnaire and modified Beck Depression Inventory II were administered to 700 students, selected by multi-stage stratified sampling, from the main campus of Jimma University. Descriptive statistics, simple and multiple logistic regression models were used to analyze possible confounders. Presence of crude association between the dependent and independent variables was detected by bivariate logistic regression analysis. Variables with p value < 0.25 in bivariate analysis were analyzed by multivariable logistic regression to exclude the confounders. Adjusted odd ratios with 95%CI were computed to examine depression symptoms and other independent variables as predictors of RSB.

**Results:**

RSB were reported by 30.2% students. Out of 222 (33.6%) students with depression symptoms 105 (47.3%) reported RSB. Students with moderate depression symptoms are nearly two times more likely to experience risky sexual behavior than students with no depression symptoms (AOR 1.9, 95% CI: 1-3.1). Students with severe depression symptoms are nearly two and half times more likely to experience RSB than students with no depression symptoms counterparts (AOR 2.6, 95%CI: 1.3- 5.1).

**Conclusion:**

RSB were high among students with depression symptoms in the main campus of Jimma University. To help students overcome the challenges, recommendation was given for concerted action from the University, governmental and NGO, and the surrounding community to establish support services and various reproductive and mental health awareness programs within the campus.

## 1. Introduction

The transition from adolescence to adult hood is characterized by sharp boundaries. It is a challenging time with regard to psychosocial development [1, 2]

Physiological changes during adolescence may explain young peoples' motivation to explore a range of different activities and experiment in high-risk behaviors such as substance abuse and risky sexual activity [3]. Young adulthood is a challenging time with regard to psychosocial development. The struggle to find and test one's own identity, to “fit in,” and to build self-esteem often takes place through experimentation in different areas of behavior, including sexual relation [4, 5]

Adolescents and young adults have increased interest in the opposite sex, highly concerned with physical and sexual attractiveness, and are frequently changing relationships. Besides, risk takers are more likely to make decisions about the future without adequately considering the consequences [6]. Young age is both a period of opportunity as well as a time of vulnerability- a time of experimentation with new ideas and options and marked with vulnerability to health risk and those related to unsafe reproductive health outcomes [7]

Young age is also a critical developmental period when many youth begin to define and clarify their sexual values and start to experiment with sexual behaviors. Most of these youth are students and they are also at a high risk for unsafe sexual behaviors and problems like HIV/AIDS or STI, unwanted pregnancy, abortion, poor school performance, high school dropout rate, psycho-social problems, conduct disorder, divorce, and economic problems [8, 9]

Students of higher institutions are the backbone of social development, asset the society, and are change agents on which the future fates of any nation relay. It is also clear that this group is on the way of transforming to adulthood; filled with ambition; and building their future academic and social career. Neglecting their sexual and reproductive health can lead to high socio-economic consequences both immediately and in the years ahead [10, 11]

Risky sexual behaviors are behaviors that include engaging in sexual activity from an early age, inconsistent use of condoms during sexual intercourse, unprotected sexual intercourse, having sex with commercial sex workers, and the tendency to have multiple sexual partners [3, 12–14].

Risky sexual behaviors expose young adults to sexually transmitted disease like Human Immunodeficiency Virus (HIV), to unconsented (forceful sex {rape}), unplanned pregnancy, and abortion which can lead to death and disability [15].

Young people of 10-24 years of age make up one-quarter of the world's population. Furthermore, according to the 2012global Acquired Immunodeficiency (AIDS) report, the same age group made up 42% of all new HIV infections in 2010. As to the setting, according to the 2012 United Nations Program on HIV and AIDS (UNAIDS) Global report, Sub-Saharan Africa remains the most severely affected, where nearly one in every 20 adults (4.9%) are living with HIV, which accounts for 69% of the people living with HIV worldwide [3, 16].

The onset of mental health problems and risky sexual behavior both reach a peak in young adulthood [5]. Poor mental health has strong associations with other health concerns in this age group, substance abuse, and violence [4, 5].

Depression in young adults is a serious public health problem and the source of immense human suffering. It disrupts the person's life during a critical period for learning and social development. University students are often undergoing role transitions—such as moving away from the family home for the first time, residing with other students, and experiencing reduced adult supervision; these changes may increase the risk of depression [17, 18]

One of the most common mental health problems faced by youths is depression, with estimates of lifetime prevalence over the course of adolescence ranging from 15% to 20%. High rates of sub threshold depressive symptoms among youths have also been reported [19].

While sexual risk behaviors and STI are risk factors for depression, depression also may increase susceptibility to risk sexual behaviors and infection [20]. Depression may impair cognitive function, memory, decrease impulse control, contribute to psycho-social impairment including emotional reactivity in peer relationships, reduce motivation, and increase fatalism. These depression-related effects may inhibit clear perception of the consequences risk sexual behaviors and the ability to prevent risk behavior [21].

Research has found depressive symptomatology among youth to be associated with earlier sexual debut, higher numbers of lifetime sexual partners, concurrent, multiple and casual sexual partnerships, substance use at last sex, pregnancy, non-use of contraception, perceived barriers to condom use, and having more risky partners [22, 23].

It is reasonably possible to assume that university students are educated, inspirational, flourished with information, and able to practice upon the information they receive and as a result, they are among the low risk population. Nevertheless, practical observation showed that for many campus students the opposite appears to be the case [24, 25].

## 2. Materials and Methods

### 2.1. Study Area and Period

The study was conducted in the main campus of Jimma University, April 5 to 20, 2016. Main campus has currently four colleges with total of thirty departments: College of Health Science (medicine, pharmacy, medical Laboratory, anesthesia, dentistry, nursing, midwifery, health officer, and environmental health departments), College of Natural and Computational Science (mathematics, sport, chemistry, physics, biology, statistics and information science departments), College of Social Science and Humanities (geography, history, Amharic, English, Afan Oromo, sociology, music, Oromo folklore, social work, and psychology departments), and College of Law and Governance (Law and Governance departments). There were 6,155 regular undergraduate students enrolled during the academic year.

### 2.2. Study Design

Institution based quantitative cross-sectional study was conducted.

### 2.3. Population

#### 2.3.1. Source of Population

All regular undergraduate Jimma University main campus students.

#### 2.3.2. Study Population

Selected regular undergraduate main campus students who are enrolled from 1^st^ year to 5^th^ year in 2015/16 academic calendar.

### 2.4. Eligibility Criteria

#### 2.4.1. Inclusion Criteria

All regular undergraduate Jimma University main campus students who are enrolled in 2015/2016 academic calendar.

#### 2.4.2. Exclusion Criteria

Students who had accidental illness during data collection period which make them incapable of participating in the study.

### 2.5. Sample Size Determination

The sample size was determined by single population proportion formula by assuming prevalence of risky sexual behavior rate of 31.4%, according to the study done in Arbaminch university [14] with 5% margin of error and 95% confidence interval of certainty (alpha = 0.05).

The actual sample size for the study was computed using the following formula;

(1) $$\begin{array}{c} n=\frac{{\left(z\alpha /2\right)}^{2}P\left(1-p\right)}{d^{2}} \end{array}$$

where

n = Sample size

z = critical value 1.96

*α* /2= confidence level

P= prevalence of risky sexual behavior at Arbaminch university students = 0.314.[14]

d= margin of error=0.05 (5%)

Therefore the value of n is calculated as follows:

(2) n1.962 x 0.3141−0.3140.052=330.99  approximately  331

Since the total population is less than ten thousands correction formula was used to get the desired sample size

(3) nf=n1+n/Nnf=3311+331/6155=314.1  approximately  315

Since the sampling is multistage, design effects of 2 were taken, 315 x 2, then it becomes 630.

Finally with addition of 10% non-response rate the required sample size becomes 700.

### 2.6. Sampling Technique and Procedures

Multistage stratified sampling technique was used to select the study participants. All four colleges were included. Stratification has been done on department and year of study level. From the thirty departments in the four colleges, eleven departments were selected by simple random sampling lottery method. From these departments, after proportionate allocation of the students for each year of study (first year to fifth year), simple random sampling technique was used to select 700 students, using the enrollment register as a frame.

### 2.7. Variables

*Dependent Variables*

- Risky sexual behavior (presence or absence)

*Independent Variable*

*Socio Demographic Economic Variables*

- Age
- Sex
- Religion
- Ethnicity
- Marital status
- Pocket money
- Place of Growing
- Participation in Religious Education

*Educational Related Factors*

- Department/ field of study
- Year of study

*Mental Health Factor*

- Depression

*Substance Related Factors*

- Khat use
- Alcohol Consumption

*Family Related Factors*

- Living Arrangements
- Educational level of parents
- Discussion with parents on sexual matters

*Factors Influencing for Acquirement of Risky Sexual Behavior*

- Watch pornographic movie
- Attending night clubs
- Peer pressure to engage into risky sexual behaviors

### 2.8. Data Collection Instruments and Procedures

A structured, self-administered questionnaire consisting of five different sub-sections was used. The questionnaire has socio-demographic, depression symptoms, Khat chewing/use, Alcohol consumption, and Risky sexual behaviors sections. The research tool was developed after extensive literature search. For risky sexual behavior assessment tool, face validity test by three independent experts on the field was performed and reliability test was done with Cronbach alpha result of 0.78. Beck Depression Inventory- II (BDI-II) was used to evaluate severity of depression symptoms with good psychometric property.

The questionnaire was prepared in English, then translated into the local languages, Amharic and Afan Oromo and back translated to English by language experts, so as to ensure its consistency. Finally, the Amharic and Afan Oromo versions of the questionnaires were used to collect data based on the respondent's language preference.

The data collectors were five BSc psychiatry nurses. Data collectors and supervisors were trained for two days by the principal investigator on the objective, purpose of the study, and data collection procedure.

### 2.9. Data Quality Management

Regular supervision was made by the supervisor and the principal investigator to ensure that all necessary data are properly collected. Each day of data collection, the filled questioners were cheeked manually first for completeness and consistency then the collected data were processed timely and entered from a paper onto computer twice. Pre-test was conducted before the main study on 35 students (5% of the sample size) at Jimma University college of Agriculture and Veterinary Medicine to identify impending problems on data collection tools. Data collected in the pre-test were not included in the analysis as part of the main study.

### 2.10. Data Processing, Analysis and Interpretation

The collected data were checked manually for completeness and consistency, cleaned, coded and entered, into Epi-data version 3.1, and exported to SPSS version 20 statistical software for analysis. Descriptive statistics were done to summarize the dependent and independent variables. Logistic regression model was used to analyze the predictors of risky sexual behavior. Bivariate logistic regression was done and variables with p-value less than or equal to 0.25 were entered into multivariate logistic regression, then variables with p- value <0.05 at 95% confidence interval were considered as statistically significant association with the outcome variable.

### 2.11. Operational and Term Definitions

*Sex Contact*. Voluntary or involuntary sexual intercourse.

*Sexual Experience*. The experience of having sexual intercourse.

*Risky Sexual Behavior (RSB)*. Not using condom or inconsistent use of condoms, or having multiple sexual partner or early initiation of sex or sex with commercial sex workers. Participants who are engaged into at least one of the above behaviors were considered as having risky sexual behavior and those who were not engaged into non-of the above behaviors were considered as not having risky sexual behavior[3, 25].

*Not Using Condom*. Never used condom on sexual intercourse until the survey.

*Inconsistent Use of Condom*. Fail to use condom at least ones during sexual intercourse until the survey.

*Having Multiple Sexual Partners*. Participants who had two or more sexual partners until the survey.

*Early Initiation of Sex*. Participants who start sex before age 18 years.

*Sex with Commercial Sex Workers*. Sexual act with those who do sex for exchange of money until the survey.

*Depression Symptoms*. Measured by Beck Depression Inventory- II (BDI-II). The scores are interpreted as 0 to 13 indicating no or minimal, 14 to 19 mild, 20 to 28 Moderate, and 29 to 63 severe depression symptoms [26].

*Substance*- in this study includes Khat and Alcohol.

*Alcohol Consumption*. It was measured by AUDIT. AUDIT score of 1–7 indicates social drinking, Score of 8–15 “hazardous drinking”, Score of 16-19 “harmful drinking” Score of 20 or above probable alcohol dependence. Participants with AUDIT score of eight or more will be used to define probable ‘alcohol use disorder [27].

*Khat Use*. Measured by- Life time Prevalence of Khat use is the proportion of student who had ever used or chewed Khat and Current Prevalence of Khat use is the proportion of students who use or chew currently and have chewed within month of data collection.

### 2.12. Ethics and Consent

The proposal of this study was reviewed and approved by Institutional Review Board (IRB) of Jimma University. Written consent was obtained from participants after explaining the purpose of the study. Participants were assured that their names will not be stated; data were kept confidential & anonymous and used only for research purpose. Study participant with problem related to Alcohol consumption, depression symptoms, and having suicidal thought are linked to nearby mental health service providing facility.

## 3. Results

The questionnaires were completed by 660 students.

### 3.1. Socio Demographic Characteristics of Participants

Majority of the respondents were males (n=422, 63.9%), unmarried (n=568, 86.1%) with mean age of 21. 29 (SD + 1.92) years. They were predominantly Orthodox Christians (n=269, 40.8%) and Muslims (n=201, 30.5%). Maximum representation was by Oromo and Amhara ethnic tribes (n=352, 53.3%; n=155, 23.5%). Most of them were from the College of Health Science (n=273, 41.4%), pursuing first year of study (n=208, 31.5%). See Table 1.

### 3.2. Prevalence of Risky Sexual Behavior

The prevalence of RSB among Jimma University main campus regular undergraduate students was 30.2% (n=199).

### 3.3. Factors Influencing the Acquirement of Risky Sexual Behavior

Majority of the respondents did not watch porn movies (n=386, 58.5%) nor attend night clubs (n=517, 78.3%). There was no peer pressure to engage in risky sexual behaviors (n=492, 74.5%), and 80.8% (n=533); students reported it was taboo to openly discuss sexual issues with parents. See Table 2.

### 3.4. Depression Symptoms

The BDI-II, which was used to identify depressive symptoms and its severity, found 29.1% (n=192) respondents had depression symptoms, among them 88 (n=13.3%) had mild depression symptoms, 81 (n=12.3%) had moderate depression symptoms, and 23 (n=3.5%) had severe depression symptoms.

### 3.5. Sexual Practice and Risky Sexual Behavior

Out of 660 students participating in the study, 35.5% (n=234) participants reported sexual intercourse, 61.1% (n= 143) of them had multiple sexual partners, and 59.8% (n=140) had sexual relation within last 1 year. Majority of these sexually active participants between 14-25 years had early sexual initiation 44.9% (n=105). Although majority of them reported use of condom (n=164, 70.1%), 29.9% (n=70) had not used condoms. Among the condom users, majority 59.8% (n=98) always used condom during coitus, whereas 24.4% (n=40) reported occasional use. Sex with commercial sex workers was reported by 5.5% (n=36), among them all are those who had reported sexual experience. See Table 3.

### 3.6. Factors Associated with Risky Sexual Behavior

Age, sex, marital status, current living condition, attending religious education, mother education attending night clubs, watching pornographic movies, peer pressure to engage in risky sexual behaviors, having depression symptoms, having alcohol use disorder showed highly significant association with risky sexual behavior (P ≤0.25) and were further analyzed by multivariate logistic regression to control the confounders.

Depression symptoms, probable alcohol use disorder, watching pornographic movie, not participating in religious education, and having peer pressure to engage in risky sexual behaviors were independent predictors of risky sexual behavior.

Students with moderate depression symptoms are nearly two times more likely to indulge in risky sexual behavior than students with no depression symptoms (AOR 1.9, 95%CI: 1.1-3.1). Also students with severe depression symptoms are nearly two and half times more likely to experience risky sexual behavior than students with no depression symptoms (AOR 2.6, 95%CI: 1.3- 5.1). Having alcohol use disorder students are nearly three times more likely to experience risky sexual behavior than the non-drinker counterparts (AOR 2.9, 95%CI: 1.4-6.1). Students who chewed Khat 2-4 times a month are approximately three times more likely to experience risky sexual behavior than the non-chewer counterparts (AOR:2.8, 95%CI: 1.1-7.7). Students who chewed Khat 2-3 times a week are approximately four and half times more likely to experience risky sexual behavior than the non-chewer counterparts (AOR 4.3,95% CI: 1.1-17.6). Students who didn't participate in religious education are nearly two times more likely to experience risky sexual behavior than their counterpart (AOR 1.9, 95%CI: 1.1-3.2). Students who watched pornographic movies are approximately four times more likely to experience risky sexual behaviors (AOR 4.1, 95%CI: 2.6-6.5). Students who had peer pressure to engage in risky sexual behaviors are approximately one and half times more likely to experience risky sexual behaviors (AOR 1.6, 95%CI: 1.1-2.7). See Table 4.

The level of depression symptoms in students, among other factors, was found to be significantly associated with involvement in risky sexual behaviors. Thus, students with moderate depression symptoms (AOR 1.9, 95%CI: 1.1-3.1), severe depression symptoms (AOR 2.6, 95%CI: 1.3- 5.1), alcohol use disorder (AOR 2.9, 95%CI: 1.4-6.1), chewing Khat (AOR 2.8, 95%CI: 1.1-7.7), avoiding religious education (AOR 1.9, 95%CI: 1.1-3.2), watching pornographic movies (AOR 4.1, 95%CI: 2.6-6.5), and peer pressure for risky sexual practices (AOR 1.6, 95%CI: 1.1-2.7) were more likely to indulge in risky sexual behaviors. See Table 5.

## 4. Discussion

This study was planned to assess the association of risky sexual behavior with depression symptoms among Jimma University main campus students, Ethiopia.

The prevalence of risky sexual behaviors was 30.2 %; similar findings were reported from Arbaminch University 31.4% [14], but much lower compared to Haramaya University students, where 65.8% of the participants had practiced at least one of the risky sexual behaviors. In the study done at Haramaya University, the prevalence of risky sexual behavior was calculated from only those students who were sexually active, unlike the case in this study where the prevalence was calculated from all the study participants [25] but much higher than Humera secondary school which was 13.7% [13]. College life gives students sense autonomy. Being away from parental constraints and influenced by friends, students could go in pursuit of forbidden pleasures such as substance use and seek sexual gratification from commercial sex workers.

The study revealed 44.9% students had early sexual debut (<18years); similar findings were reported in Haramaya University 43.5 % [25] but higher than the findings from Bahir Dar University 24.3 % [15]. The findings are comparatively lower than those of the report from Cameroon University 55% [28]. This is another nation, culturally different. Lower findings in studies done six years ago at Jimma University and Bahir Dar University 75.6% and 72.8% respectively are explained by the obvious time gap and the media interventions on sexual health [10, 15].

There was an increase in students having multiple sexual partners in the current study (61.1%), overtaking the results reported six years ago in the same University 28.3% [10]. and other research findings from Bahir Dar University 42.7%, Jigjiga university 30.1%, Mekelle University 47.4 %, and tertiary institution in Nigeria 48.2% [15, 17, 29, 30]. The growing influence of social media and covert peer pressure in the campus may be promoting sexual promiscuity.

A little more than one-fourth of the students (29.9%) had never used condom and the rest (70.1%) used condoms during casual sex. But only a little more than half of them (59.8%) always used condom whereas 24.4% used occasionally and the remaining 15.8% rarely used. The study done in Bahir Dar university reported similar findings for inconsistent condom use and never use of condom 36% and 25% respectively [15]. Our findings are comparable to the study conducted in Mekelle University in which among those who used condom 50.5% were inconsistent condom users [30]. In Madagascar University 57.6% of the respondents used condom inconsistently [31]. But our finding was higher than that of the study done in Kenya, Nairobi University students and Colombia with inconsistent condom use by 27.4%, 33.7% respondents respectively [23, 32]. This disparity may be explained by the difference in culture of using condom between the two countries.

Sex with commercial sex workers was reported by 5.5% students compared to 8.8% from Arbaminch University, 7.4% Bahir Dar University, 31.9% Haramaya University, 16.3 % Jigjiga University, and 8.8% Gondar University respectively [14, 15, 25, 29, 33].

The risk of engaging in risky sexual behaviors was four times higher among those who watched pornographic movies than their counterparts, consistent with studies from Mizan-Tepi University indicating three times increased risk for risky sexual behaviors [34]. This is also supported by study done in Bahir Dar University and Jimma University which found the risk was two times higher than those who were not exposed to porn movies respectively [10, 15]. Pornographic movies can excite the students and put people at risk for unsafe sexual activities.

Peer pressure in the campus was another risk factor which exposed the students for risky sexual behaviors two times more than those who did not experience peer interference; a similar finding was revealed in a previous study in the same University [10]. At Humera secondary school, peer pressure increased the risk by three times [13]. Peers can be a negative influence on students with low self-esteem. The desire to be in the ‘in –group' and being away from parental control are other reasons for many students to try out risky behaviors, often overlooking the painful consequences.

Students who participated in religious education were two times less likely to involve in risky sexual behaviors; the study among students of Humera secondary school also found religious education being a protective factor against exposure to risky sex. [13]

Risky sexual behaviors were reported by 30.2% students. Out of 222 (33.6%) students with depression symptoms 105 (47.3%) reported risky sexual behaviors. Moreover students with severe depression symptoms were two and half times more likely to engage in risky sexual behaviors than students with no depression symptoms (P value of 0.003); this finding is in line with the study done among Nairobi University students, Kenya [23] and US middle and high school Students [35]. The possible explanations can come from Beck's cognitive theory of depression, which describes that negative feelings and thoughts play a central role in how people feel about themselves, which ultimately influences the behavior in which they engage. Such negative thoughts and cognitive distortions can deter rational decision-making and allow emotions to influence behavior. Thus, depressive symptoms may not support healthy decision making and subsequent healthy behavior, including safe sexual decision making and safe sex behavior [36]. Another explanation can be individuals with depressive symptoms may also use sexual risk behaviors as part of a coping response to their depressive symptoms and depression can unearth unbearable feelings that many people try to escape and patients attempt to seek relief from emotional pain which exposes them to self-destructive behaviors such as substance use and indiscriminate sex. [37].

Another valid finding in this study, which has been supported by other literature from Uganda, Ireland and USA, was the higher risk for risky sexual behaviors among students with probable alcohol use disorders [38–40] Dependence on mind altering substances including alcohol, alters sound thinking and social judgment and therefore less likely to enact protective behaviors. Moreover the link between alcohol disorder and depression symptoms among these students may exist. The possible reason alcohol use increases risk of practicing risky sexual behavior in all studies may be due to the fact that alcohol interferes with judgment and decision making capacity which led the user to be involve in various risky sexual behaviors.

The use of Khat was another risk factor in this study which exposed students to risky sexual behaviors. In this study students who chewed Khat 2-4 times a month and 2-3 times a week were at higher risk to engage in to risky sexual behavior than those who never chewed, which has been confirmed by studies at Universities in Arbaminch, Bahir Dar and Haramaya [14, 15, 25]. Khat interferes with cognitive capacity and interferes with judgment and in some cases also increases sexual desire.

## 5. Conclusion

The prevalence of risky sexual behavior and depression symptoms among Jimma university main campus students was high and positively associated. Moreover, substance use, watching pornographic movie, having peer pressure to engage in risky sexual behaviors, and not participating in religious educations have found to increase the risk of experiencing risky sexual behavior.

## Figures and Tables

**Table 1:** Socio demographic, economic, and academic and parent's characteristics of Jimma University students, April, 2016.

	Characteristics	Frequency (n=660)	Percentage

Sex	Male	422	63.9
	Female	238	36.1

Age	18-20	273	41.4
	21-23	305	46.2
	>=24	82	12.4

Marital status	Married	73	11.1
	Unmarried	587	88.9

Ethnicity	Oromo	352	53.3
	Amhara	235	23.5
	Gurage	52	7.9
	Tigre	39	5.9
	Yem	28	4.2
	Others	34	5.2

Religion	Orthodox	269	40.8
	Islam	202	30.5
	Protestant	156	23.6
	wakefeta	21	3.2
	Catholic	8	1.2
	Others	5	0.8

Living Residence before joining university	Rural	270	40.9
	Urban	390	59.1

Living arrangement	Living with parent	107	16.2
	Living away from parent	553	83.8

Current living residence	In dormitory	494	89.3
	Outside dormitory in rented house	59	10.7

Participation in religious education	Yes	539	81.7
	No	121	18.3

Average Monthly pocket money(birr)	None	46	7.0
	<100	85	12.8
	100-299	153	23.2
	300-499	241	36.5
	≥ 500	135	20.5

College	Health science	273	41.4
	Law and governance	155	23.5
	Social science and humanities	150	22.7
	Natural and computational science	82	12.4

Year of study	Year I	208	31.5
	Year II	170	25.8
	Year III	128	20.9
	Year IV	90	13.6
	Year V	54	8.2

Mother's educational status	Illiterate	165	25.0
	Primary school	281	42.6
	Secondary school	129	19.5
	University/ collage	85	12.9

Father's educational status	Illiterate	108	16.4
	Primary school	231	35.0
	Secondary school	202	30.6
	University/ collage	119	18.0

Other ethnicity= Wolayta, Sidama, Kafa, Hadiya, and Silte.

Other religion = Giova witness, Adventist, atheist.

**Table 2:** Factors influencing acquirement of risky sexual behavior among Jimma University students, April, 2016.

Characteristics	Frequency (n=660)	Percentage

Watching pornographic movie		
Yes	274	41.5
No	386	58.5

Attending night clubs		
Yes	143	21.7
No	517	78.3

Peer pressure to engage in risky sexual behaviors		
Yes	168	25.5
No	492	74.5

Discussion with parents on sexual matters		
Yes	127	19.2
No	533	80.8

**Table 3:** Participant's sexual practices and risky sexual behavior of Jimma University students, April, 2016.

Variables	Characteristics	Frequency	Percentage (100%)

Sexual experience	No	426	64.5
	Yes	234	35.5

Sex in the last 12 months (sexually active)	Yes	140	59.8
	No	94	40.2
	Total	234	100

Number of sexual partners	No	426	64.5
	1	91	38.9
	>1	143	61.1
	Total	234	100

Age at first sexual intercourse	>=18	129	55.1
	<18	105	44.9
	total	234	100

Condom use	Yes	164	70.1
	No	70	29.9
	Total	234	100

Frequency of condom use	Always	98	59.8
	Occasionally	40	24.4
	rarely	26	15.8
	total	164	100

Sex with commercial sex workers	yes	36	5.5
	No	624	94.5
	total	660	100

**Table 4:** Bivariate analysis of factors associated with risky sexual behavior among Jimma university students, April 2016.

	Characteristics	Risky sexual behavior		COR (CI-95%)	P value
		Yes	No		
Sex	Male	149(74.9%)	273(59.2%)	2.0(1.4-2.9)	< 0.001∗
	Female	50(5.1%)	188(40.8%)	1	

Age	18-20	71(26.0%)	202(74.0%)	1	
	21-23	92(30.2%)	213(69.8%)	1.2(0.8-1.7)	0.268
	>=24	36(43.9%)	46(56.1%)	2.2(1.3-3.7)	0.002∗

Marital status	Married	46(63.0%)	27(37.0%)	1.4(0.8-2.3)	0.179∗
	Unmarried	415(70.7%)	199(29.3%)	1	

Living residence before joining university	Rural	88(32.6%)	182(67.4%)	1.2(0.8-1.7)	0.256
	Urban	111(28.5%)	279(71.5%)	1	

Living arrangement	Living with parent	37(34.6%)	70(65.4%)	1.2(0.8-1.9)	0.276
	Living away from parent	162(29.3)	391(70.7%)	1	

Current living condition	In dormitory	22(37.3%)	37(62.7%)	1.5(0.8-2.6)	0.156∗
	Outside dormitory in rented house	140(28.3%)	354(71.7%)	1	

Participation in religious education	Yes	137(25.4%)	402(74.6%)	1	
	No	62(51.2%)	59(4.8%)	3.0(2.0-4.6)	< 0.001∗

Monthly Pocket money(birr)	None	14(30.4%)	32(69.6%)	0.8(0.4-1.7)	0.651
	<100	29(34.1%)	56(65.9%)	1(0.5-1.7)	0.995
	100-299	52(34.0%)	101(66.0%)	1(0.6-1.6)	0.987
	300-499	58(24.1%)	183(75.9%)	0.6(0.3-1.1)	0.388
	≥500	46(34.1%)	89(65.9%)	1	

Collage	Health science	78(28.6%)	195(41.4%)	1	
	Law and governance	46(30.7%)	104(69.3%)	1.2(0.8-1.9)	0.282
	Social science and humanities	23(28.0%)	59(72.0%)	1.1(0.7-1.7)	0.651
	Natural and computational science	52(33.5%)	103(66.5%)	0.9(0.5-1.6)	0.927

Year of study	Year I	54(26.0%)	154(74.0%)	1	
	Year II	48(28.3%)	122(71.8%)	1.1(0.7-1.7)	0.620
	Year III	46(33.3%)	92(66.7%)	1.4(0.8-2.2)	0.139
	Year IV	27(30.0%)	63(70.0%)	1.2(0.7-2.1)	0.472
	Year VI	24(44.4%)	30(55.6%)	2.2(1.2-4.2)	0.009∗

Mother education level	Illiterate	56(33.9%)	109(66.1%)	1	
	Primary school	85(30.2%)	196(69.8%)	0.8(0.5-1.2)	0.419
	Secondary school	34(26.4%)	95(73.6%)	0.6(0.4-1.1)	0.162∗
	University/ collage	24(28.2%)	61(71.8%)	0.7(0.4- 0.3)	0.360

Father education level	Illiterate	34(31.5%)	74(68.5%)	1.1(0.6-2.1)	0.536
	Primary school	68(29.4%)	163(70.6%)	1.0(0.6-1.7)	0.739
	Secondary school	64(31.7%)	138(68.3%)	1.2(0.7-1.9)	0.457
	University/ collage	33(27.7%)	86(72.3%)	1	

Watch pornographic movie	Yes	135(49.3%)	139(50.7%)	4.8(3.4-6.9)	< 0.001∗
	No	64(16.6%)	322(83.4%)	1	

Attending night clubs	Yes	82(57.3%)	61(42.7%)		
	No	117(22.6%)	400(77.4%)	4.5(3.1-6.7)	< 0.001∗

Peer pressure to engage into risky sexual behavior	Yes	80(47.6%)	88(52.4%)	2.8(1.9-4.1)	< 0.001∗
	No	119(24.2%)	373(75.8)	1	

Discussion with parents on sexual matters	Yes	40(31.5%)	87(68.5%)	1.0(0.7-1.6)	0.713
	No	159(29.8%)	374(70.2%)	1	

Level of depression symptoms (BDI score)	No/minimal(0-13)	108(23.1%)	360(76.9%)	1	
	Mild (14-18)	43(48.9%)	45(51.1%)	1.3(0.8-2.1)	0.158∗
	Moderate (19-28)	35(43.2%)	46(56.8%)	3.4(2.1-5.6)	< 0.001∗
	Severe (29-63)	13(56,5%)	10(43.5%)	3.4(2.0-5.7)	< 0.001∗

Alcohol	Had probable alcohol use disorder	44(73.3%)	16(26.7%)	7.8(4.3-14.3)	< 0.001∗
	Had no alcohol use disorder	155(25.8%)	445(74.2%)	1	

Frequency of Khat use	Never chewed	119(24.7%)	362(75.3%)	1	
	Monthly or less	45(36.3%)	79(63.7%)	1.7(1.1-2.6)	0.100
	2-4 times a month	19(61.3%)	12(38.7%)	4.8(2.2-10.2)	< 0.001∗
	2-3 times a week	13(68.4%)	6(31.6%)	6.5(2.4-17.7)	< 0.001∗
	4 or more times a week	3(60.0%)	2(40.0%)	4.5(0.7-27.6)	0.999

∗ Variables which were associated with risky sexual behavior in the bivariate analysis

BDI- Beck Depression Inventory

AUDIT- Alcohol Use Disorder Identification Test

1-Reference.

**Table 5:** Multivariate analysis of factors associated with risky sexual behaviors among Jimma University students, April, 2016.

variables	Characteristics	Risky sexual behaviors		COR	AOR (CI=95%)	P- value
				(CI-95%)		
		Yes	No			

Participation in religious education	*Yes*	137(25.4%)	402(74.6%)	1		
	*No*	62(51.2%)	59(4.8%)	3.0(2.0-4.6)∗	1.9(1.1-3.2)	0.010∗∗

Watch pornographic movie	Yes	135(49.3%)	139(50.7%)	4.8(3.4-6.9)∗	4.1(2.6-6.5)	< 0.001∗∗
	No	64(16.6%)	322(83.4%)	1		

Peer pressure to engage into risky sexual behavior	Yes	80(47.6%)	88(52.4%)	2.8(1.9-4.1)∗	1.6(1.1-2.7)	0.024∗∗
	No	119(24.2%)	373(75.8)	1		

Level of depression symptoms (BDI)	No/minimal(0-13)	108(23.1%)	360(76.9%)	1		
	Mild (14-18)	43(48.9%)	45(51.1%)	1.3(0.8-2.1)∗	0.9(0.3-1.5)	0.705
	Moderate (19-28)	35(43.2%)	46(56.8%)	3.4(2.1-5.6)∗	1.9(1.1-3.1)	0.049∗∗
	Severe (29-63)	13(56,5%)	10(43.5%)	3.4(2.0-5.7)∗	2.6(1.3- 5.1)	0.003∗∗

Alcohol	Had probable alcohol use disorder	44(73.3%)	16(26.7%)	7.8(4.3-14.3)∗	2.9(1.4-6.1)	0.004∗∗
	Had no alcohol use disorder	155(25.8%)	445(74.2%)	1		

Frequency of Khat use	Never chewed	119(24.7%)	362(75.3%)	1		
	Monthly or less	45(36.3%)	79(63.7%)	1.7(1.1-2.6)	1.1(0.6-2.0)	0.536
	2-4 times a month	19(61.3%)	12(38.7%)	4.8(2.2-1.2)∗	2.8(1.1-7.7)	0.042∗∗
	2-3 times a week	13(68.4%)	6(31.6%)	6.5(2.4-17.7)∗	4.3(1.1-17.6)	0.040∗∗
	4 or more times a week	3(60.0%)	2(40.0%)	4.5(0.7-27.6)	2.1(0.1-25.9)	0.519

∗ Variables which were associated with risky sexual behavior in bivariate analysis

∗∗ Variables which were independently associated with risky sexual behavior in multivariate analysis

1- Reference.

## Data Availability

The data used to support the findings of this study are available from the corresponding author upon request. Data underlying the findings of this paper will be publicly available.

## Supplementary Materials

Supplementary Materials The supplementary material is a data set. All the research article result is generated from that data set. The data set includes all the variables with corresponding respondents response. The analysis results were also generated from that data set. Some variables are coded in different variables for description purpose.
